# A Single-Boundary Accumulator Model of Response Times in an Addition Verification Task

**DOI:** 10.3389/fpsyg.2017.01225

**Published:** 2017-07-18

**Authors:** Thomas J. Faulkenberry

**Affiliations:** Department of Psychological Sciences, Tarleton State University Stephenville, TX, United States

**Keywords:** mental arithmetic, format effects, accumulator model, shifted Wald distribution

## Abstract

Current theories of mathematical cognition offer competing accounts of the interplay between encoding and calculation in mental arithmetic. Additive models propose that manipulations of problem format do not interact with the cognitive processes used in calculation. Alternatively, interactive models suppose that format manipulations have a direct effect on calculation processes. In the present study, we tested these competing models by fitting participants' RT distributions in an arithmetic verification task with a single-boundary accumulator model (the shifted Wald distribution). We found that in addition to providing a more complete description of RT distributions, the accumulator model afforded a potentially more sensitive test of format effects. Specifically, we found that format affected drift rate, which implies that problem format has a direct impact on calculation processes. These data give further support for an interactive model of mental arithmetic.

## Introduction

Response times (RTs) have long held a privileged status as one of the primary behavioral measures in cognitive research (Luce, 1986). Their role in inferring mental processes has become so ubiquitous that the justification of their use is rarely questioned. As Luce (1986) himself put it, “we surely do not understand a choice process very thoroughly until we can account for the time required for it to be carried out” (p. vii). This is particularly evident in the study of mathematical cognition, and in particular, the study of mental arithmetic processes. Since the seminal work of Groen and Parkman (1972), RTs have provided the primary behavioral signatures used to theorize about the nature of mental calculation. The purpose of the present paper is to extend this work and weigh in on a long-standing debate concerning the independence of encoding and calculation. We accomplish this by fitting distributions of RTs in a mental addition task with a mathematical model known as a shifted Wald distribution and subsequently assessing the effects of format and problem size manipulations on the parameters of these distributions.

### Models of mental arithmetic

A central question in mathematical cognition concerns the nature of the processes involved in mental arithmetic. Over the years, several competing models of mental arithmetic have been proposed. While most models share a serial architecture of encoding, calculation (which may include retrieval), and production (Ashcraft, 1992), these competing models differ with respect to the proposed independence of these stages. The *abstract code model* (McCloskey, 1992; McCloskey et al., 1992; McCloskey and Macaruso, 1995) proposes separate encoding, calculation, and production modules that communicate via an abstract semantic representation. Each module is specialized for a particular type of input; that is, there are separate encoding modules for verbal numerals (e.g., “four”) and Arabic numerals (e.g., “4”). For example, consider the problem 3 + 4. According to the abstract code model, this problem would be solved by first entering through a comprehension module, specialized for the type of stimulus (in this case, Arabic numerals). After this initial encoding, the problem would then be converted to an amodal, semantic representation (an “abstract code”). Calculation (e.g., retrieval) would operate on this abstract code. The result of the calculation (the answer 7, still in the form of an abstract representation) would then feed into a production module, specialized for the type of production required in the task (either verbal or Arabic).

On the other hand, the *triple code model* (Dehaene, 1992; Dehaene and Cohen, 1995) proposes three separate modules, each specialized for a specific type of representational format: an analog magnitude representation (e.g., a mental number line), an auditory/verbal module, and a visual Arabic numeral module. The triple code model differs from the abstract code model in that calculation and production occur within each module. For comparison, consider again our example 3 + 4. In the triple code model, this problem would be input into one of two modules, each specialized for the type of input code (either an auditory-verbal word frame or a visual-Arabic number form). Calculation would then take place within one of these modules, depending on the nature of the problem. As an example, a retrieval-based calculation to a visually-presented problem (e.g., “3 + 4”) could proceed by first transcoding the input to the auditory-verbal word frame, where the appropriate arithmetic fact could then be retrieved and then the verbal answer produced (e.g., retrieving the answer as “three plus four equals seven” and then verbally producing the answer “seven”).

While the abstract code model and the triple code model differ with respect to the issue of functional vs. representational modularity, they do share a fundamentally *additive* architecture. That is, any performance differences related to problem format (e.g., faster RTs for problems written in Arabic digits compared to words) simply reflect processes related to encoding. In the context of the abstract code model, such performance differences would be explained as the cost of converting a specific stimulus type (digits or words) into an amodal, abstract semantic representation that can be further fed into an appropriate calculation mechanism. In the context of the triple code model, these performance differences would reflect a cost of converting from one representational format (e.g., verbal, word-based representation) into another format (e.g., visual, digit-based representation). Critically, both models predict that format manipulations do not interact with calculation processes.

As an alternative to such additive models of mental arithmetic, Campbell and colleagues (e.g., Campbell and Clark, 1988; Campbell, 1994; Campbell and Epp, 2004) have argued for an *interactive* architecture called the *encoding complex* model. In this model, performance differences due to manipulation of format are posited to stem from a difference in the degree of encoding-retrieval integration. For example, Arabic digits are frequently encountered in the context of calculation, and hence, strong bi-directional pathways are developed between encoding and retrieval of arithmetic facts in this format. However, number words are less frequently encountered, and hence weaker encoding-retrieval connections are formed for such inputs. While sharing some similarities with the additive models described earlier, this model differs in one critical aspect; changes in format are hypothesized to impact *both* encoding and calculation processes.

Support for such an interactive model has primarily appeared in the form of an interaction between the variables of problem format and problem size. As one of the classic “effects” in mathematical cognition, the problem size effect refers to the finding that responses for small problems (e.g., problems for which the sum of the operands is no larger than 10) are significantly faster than responses for larger problems (Ashcraft, 1992; Zbrodoff and Logan, 2005). One possible reason for the problem size effect is that large problems tend to be solved by procedural strategies, resulting in slower and more error prone responses (Campbell and Xue, 2001). While the exact mechanism underlying the problem size effect is still up for debate, the more salient finding is that the problem size effect is larger for problems presented in word format compared to digit format (Campbell and Fugelsang, 2001; Campbell and Penner-Wilger, 2006). Campbell and colleagues have argued that this interaction between problem size and format implies that format directly impacts calculation processes, providing support for the interactive model.

Nonetheless, recent research has not settled the debate regarding the independence of encoding and calculation in mental arithmetic. On one hand, some researchers have argued that people form notation-independent representations of numbers. For example, Libertus et al. (2007) recorded ERPs (event-related potentials) during a symbolic and nonsymbolic number comparison task. Adults were presented with single numbers (shown either in Arabic digit format or in nonsymbolic dot format) and asked to decide whether each was less than or greater than 15. They found that the amplitude of the P2 component (210–250 ms after stimulus presentation) increased as the distance between the number and the comparison standard decreased. Moreover, this pattern did not differ between number formats. This led Libertus et al. (2007) to conclude that number comparison proceeds via an abstract processing stage that is independent of number format. This finding mirrored previous work by Pinel et al. (2001), who used fMRI to identify regions in the parietal lobes whose activation was highly correlated with semantic properties of numbers (i.e., numerical distance), but invariant as to whether the number was presented in word or Arabic numeral format.

Similar results have been also found in behavioral experiments. For example, Ganor-Stern and Tzelgov (2008) used a size-congruity paradigm to investigate automaticity of numerical processing. In this paradigm, numbers are presented in differing physical sizes; this results in pairs of number symbols in which the physical comparison is congruent with numerical size (e.g., small 2 and large 8) or incongruent (e.g., large 2 and small 8). The usual finding is that incongruent pairs take longer than congruent pairs. This size-congruity effect (e.g., Henik and Tzelgov, 1982) is often taken as evidence for automatic processing of number magnitude. In their experiment, Ganor-Stern and Tzelgov (2008) presented Arabic speakers with number pairs written in two different notations: Arabic and Indian digits. Ganor-Stern and Tzelgov found that even in mixed pairs (one Arabic digit and one Indian digit), there was still a substantial size-congruity effect. They interpreted this result as support for the notion that both notations are automatically converted to a common representation independent of format (see also Ganor-Stern, 2009).

As mentioned earlier, evidence against this additive view of arithmetic processing has been presented by Campbell and colleagues in the form of a substantial problem size by format interaction on response times (e.g., Campbell and Fugelsang, 2001). Some researchers (e.g., Noël et al., 1997) argue that this signature on RTs does not necessarily imply that format has a direct impact on calculation processes. Noël et al. (1997) argued that such an interaction may be the result of encoding differences between digits and words that feed into the *output* stage, not the calculation stage. Finally, at least one recent study indicates that the interaction between problem size and format may not be as robust as first thought. For example, Megias and Macizo (2016) failed to find an interaction between problem size and format in a mental arithmetic task^1^. Taken together, these issues warrant further investigation of the processes in volved in mental arithmetic, as it appears that we still have more to learn about the potential interplay between encoding and calculation. In the sections below, we outline a new approach to investigating this issue, based on modeling distributions of RTs in a mental arithmetic task.

### Accumulator models of RT

Most of the studies mentioned above have employed a similar approach to analyzing the effects of experimental manipulations on RTs. Namely, for each participant, the collection of RTs for correct trials in each experimental condition is collapsed to one number, usually the arithmetic mean. This collection of means is then analyzed via an analysis of variance to determine the effect, if any, of each manipulation on RTs. Though popular, this approach is suboptimal for two reasons. First, by collapsing RTs by condition to a single numerical summary (e.g., the mean RT), we lose much information about the *distribution* of RTs. Second, this procedure is usually carried out only on *correct* trials. As such, RTs and response accuracy are analyzed separately, even though they are not necessarily independent (e.g., the speed-accuracy tradeoff, Schouten and Bekker, 1967; Wickelgren, 1977). In both cases, ease of analysis comes at the price of lost information about the original patterns of RTs.

One solution to this problem is to employ a mathematical model such as an *accumulator model*, a model for decision processes that posits a continuous uptake of noisy information that continues until the accumulated evidence exceeds a decision threshold, at which point a response is initiated (Link and Heath, 1975; Luce, 1986; Ratcliff and McKoon, 2008; Ratcliff et al., 2016). One advantage of such an approach is that instead of modeling participants' RTs in each experimental condition by a single mean RT, we can fit a model to the entire *distribution* of RTs for each participant in each experimental condition. This results in finding a set of parameters that not only *describes* the distribution mathematically, but also are indicative of the underlying cognitive processes. The advantage is that we can then directly test the effects of our experimental manipulations on the cognitive processes, not just the effects on RTs and/or errors. Hence, RTs and errors become for us a proxy to the underlying cognitive processes, not the sole object of study.

One popular example of a widely used accumulator model is the drift diffusion model of Ratcliff and colleagues (Ratcliff and Murdock, 1976; Ratcliff, 1978; Ratcliff and McKoon, 2008; Ratcliff et al., 2016), which describes a two-choice decision task as the result of such a noisy accumulation process. Specifically, a decision process is modeled as a continuous random walk {*X*_*t*_} with absorbing boundaries 0 and α. This means that the initial term of the walk *X*_0_ begins somewhere between 0 and α (i.e., 0 < *X*_0_ < α), and the walk terminates whenever *X*_*t*_ = 0 (an incorrect response) or *X*_*t*_ = α (a correct response). Moreover, the random walk terms *X*_*t*_ tend to *drift* toward one boundary or the other. That is, $\frac{d}{dt}X_{t}$ is assumed to be normal with mean γ; we refer to γ as the *drift rate*. Finally, the decision time *DT* is modeled as the first time *t* for which *X*_*t*_ hits either boundary; that is, *X*_*t*_ ≤ 0 (an incorrect response) or *X*_*t*_ ≥ α (a correct response). The total response time *RT* is then expressed as *RT* = *DT* + θ, where θ represents the nondecision component of *RT* (e.g., stimulus encoding and motor execution).

Modeling RT distributions via this diffusion process results in a set of parameters that can be mapped onto underlying latent cognitive processes. The interpretation of these parameters as indices for cognitive processes has been the subject of much investigation over the past 40 years (see Ratcliff and McKoon, 2008, for a review). Though the full Ratcliff diffusion model results in 7 such parameters (Wagenmakers et al., 2007), for simplicity we restrict our discussion to the following three parameters: α (boundary separation), γ (drift rate), and θ (nondecision time). The boundary separation parameter α represents response caution; a high value of α means that more evidence needs to accrue before a decision can be made. In other words, large values of α reflect conservative decision criteria, whereas small values of α reflect more liberal decision criteria. The drift rate parameter γ represents the *quality of information* provided by the stimulus. Larger drift rates reflect unambiguous stimuli, resulting in quicker decisions. Smaller drift rates reflect ambiguous stimuli, resulting in longer decision times. Finally, the nondecision time parameter θ reflects encoding and response processes; large values of θ reflect slower encoding and/or execution, whereas small values of θ reflect fast encoding and/or execution.

While fitting RT distributions with these parameters results in a much more detailed description of the underlying RT distributions than using the mean alone, it is not always possible to fit such a model to experimental data. For instance, one problem with the Ratcliff diffusion model is that it is not well suited to tasks with very low error rates (Anders et al., 2016). Consequently, tasks in which participants perform quite well are not fit well by the diffusion model parameters. However, an alternative accumulator model called the shifted Wald model may be well suited to such situations (Carpenter and Williams, 1995; Schwarz, 2001; Heathcote, 2004). The shifted Wald model is a model of RTs based on the Wald (1947) distribution, which represents the density of first passage times of a continuous diffusion process that drifts toward a *single* absorbing boundary. Mathematically, the probability density function of the shifted Wald model is given by:

(1) $$f(x|\alpha ,\gamma ,\theta )=\frac{\alpha }{\sqrt{2\pi {\left(x-\theta \right)}^{3}}}\cdot \exp(-\frac{{\left(\alpha -\gamma (x-\theta )\right)}^{2}}{2(x-\theta )}),$$

where α represents the height of the single response boundary, γ represents the drift rate, and θ represents a positive (rightward) shift of the entire distribution. As proxies for cognitive processes, each of these parameters has a straightforward interpretation similar to (but not quite equivalent; see Matzke and Wagenmakers, 2009) that of the Ratcliff diffusion model (Schwarz, 2001; Heathcote, 2004; Anders et al., 2016): drift rate γ reflects task difficulty via quality of information; response threshold α reflects response caution (amount of information required before response initiation), and θ reflects the nondecision time (e.g., encoding and response processes not directly related to the post-encoding decision process).

Aside from the measurement advantages of modeling RTs via distributions rather than via single point means, the shifted Wald model has further methodological and theoretical advantages in the context of mathematical cognition. First, as error rates in mental arithmetic tasks tend to be quite low, fitting RT distributions with the full Ratcliff diffusion model will be difficult. Instead, we can use the shifted Wald model to describe RT distributions for the correct responses. Further, compared to the Ratcliff diffusion model, the shifted Wald model can be fit with a relatively small number of experimental trials. For example, Anders et al. (2016) found that shifted Wald parameters can be recovered for as few as 50 observations per experimental condition. Finally, as the parameters of the shifted Wald distribution have specific cognitive interpretations, systematic variation in these parameters as a function of a stimulus manipulation (e.g., problem format) can tell us the exact locus of the effect. As described above, it is well known that problem format has an effect on RTs – problems in word format take longer to solve than problems in digit format. However, it is unclear whether this RT effect is localized to the encoding stage, the calculation stage, or both. By modeling participants' RT distributions via shifted Wald models, we can obtain a measure of how format affects each of the parameters α, γ, and θ. In the present study, we are mainly concerned with the question of whether problem format affects calculation. As such, we can make solid predictions about the effects of our manipulations on the drift rate γ, which we assume reflects the calculation process. This assumption comes from the idea that γ is a parameter related directly to a decision process, which in this experiment is a decision about the truth of a proposed addition equation. Critically, if format affects drift rate γ, this will give evidence that format also has a direct effect on calculation over and above the previously established effects of format on encoding. Such a result would favor the interactive encoding complex model over an additive model (e.g., abstract code model or triple code model), where effects of format are isolated to only the encoding stage. Note that at present, clear predictions cannot be drawn regarding the effects of our manipulations on α and θ, so for the purposes of this study, we will focus on the drift rate γ.

### The present study

There were two main goals in the present study. First, we sought to extend previous work in mathematical cognition by replicating the arithmetic verification task of Campbell and Fugelsang (2001) and applying an accumulator model (the shifted Wald model) to model the resulting RT distributions. Whereas many mental arithmetic experiments use a production task, a verification task is advantageous for this modeling approach. In addition to being the task used in Campbell and Fugelsang (2001), the verification task allows us to measure mental arithmetic processes in the framework of a two-choice decision task, which is the framework employed in most studies that employ accumulator models to study RTs. Second, we aimed to use the results of this modeling to test between two competing models: an additive model, where the stages of problem encoding and answer calculation are functionally independent and effects of problem format are isolated to the encoding stage only, and an interactive encoding-complex model, where effects of problem format are spread between both the encoding stage as well as the answer calculation stage.

## Method

### Participants

Twenty undergraduate students (15 female, mean age = 25.2 years, age range = 19–60 years) participated in this experiment in exchange for partial course credit in their psychology courses. The experiment was reviewed and approved by the institutional review board at Tarleton State University.

### Stimuli and apparatus

Our stimuli were adapted from Campbell and Fugelsang (2001). Each participant completed 288 experimental trials, consisting of four repetitions of a block of 72 single-digit addition verification problems. We manipulated both problem size and problem format. On even numbered trials, the problems were presented in word format using lower case English words (e.g., “five + seven = twelve”). On odd numbered trials, problems were presented in Arabic digit format (e.g., “5 + 7 = 12”). All problems (regardless of format) were composed of operands between 2 to 9, resulting in a set of 36 problems ranging between 2 + 2 = 4 and 9 + 9 = 18. Note that this assumes that commuted pairs such as 2 + 6 and 6 + 2 are counted as one problem. For each commuted pair, both operand orders were presented with equal frequency throughout the experiment. The order of the operands for each problem was alternated across blocks. Within each of the four blocks, each of these 36 problems was presented once in digit format and once in word format. Problem size was defined in terms of the product of operands: small problems had product operands less than or equal to 25, whereas large problems had operands greater than 25. Within each block, half of the problems of each problem size were presented with the smaller operand first; for the remaining half, the larger operand was presented first.

We further manipulated the truth value of each addition problem. Within each set of 36 problems, half were presented as true equations (e.g., “2 + 4 = 6”) and half were presented as false equations (e.g., “2 + 4 = 7”). Across all four blocks, each addition problem was tested in each format twice in a true equation and twice with a different false answer. False answers were generated pseudo-randomly to be within ±4 of the correct answer and never corresponded to either the difference or the product of the operands. Within each set of false answers, each of the numbers 4–18 (i.e., the range of true answers) occurred at least once but no more than four times.

All stimuli were presented using Superlab 5.0 (Cedrus Corporation), appearing as white characters against a black background. Responses were recorded using an RB-740 USB response box with ±2 ms timing accuracy. The experiment was run on a 21.5-inch iMac desktop computer with 1,024 × 768 screen resolution. Text was displayed in 36 point Lucida Grande font. In each problem, the two operands were separated by a single space on either side of the addition sign. The answer to be verified appeared simultaneously with the problem operands to the right and after the equal sign (e.g., 2 + 4 = 6). No other characters appeared on the screen.

### Procedure

We counterbalanced two response rules across our participants. Even-numbered participants indicated true responses by pressing the rightmost button of the response box and false responses by pressing the leftmost button. Odd-numbered participants used a reversed response mapping: they indicated true responses with the leftmost button and false responses with the rightmost button. Each participant was instructed to respond quickly but accurately.

Prior to the first block, we gave each participant a practice block, consisting of 12 trials in alternating word and digit format, using the operand 0 or 1 paired with 6 randomly selected digits ranging from 0 to 9. At the beginning of each trial, a fixation cross appeared at the center of the screen. When ready to begin, each participant initiated the presentation of the equation with a single button press. The fixation dot flashed for 1 s and was then replaced with one of the 72 addition verification problems. Timing began with the presentation of this equation and ended as soon as the participant pressed a button indicating whether the problem was true or false. After each response, feedback was given in the form of a green C (for correct trials) or red E (for incorrect trials), displayed in the center of the screen for 300ms. After feedback, the fixation cross reappeared, signaling to the participant that the next trial was ready to be initiated. After each block of 72 trials, participants were given an opportunity for a short rest. At the conclusion of the fourth block (288 trials completed), the experiment ended and participants were thanked for their participation.

## Results

Participants completed 5,760 experimental trials. Of these, 394 trials contained an incorrect response (error rate = 6.8%); these trials were removed from further analysis. To facilitate model fitting by removing potential contaminant trials, we removed any trial for which RT was below three and above six median absolute deviations (MAD) from the overall median (median RT = 1,394 ms; MAD = 633 ms) (Leys et al., 2013). This resulted in the removal of an additional 61 trials (1.1%). All subsequent modeling was done on the remaining 5,305 trials.

The general approach to modeling was as follows. First, we modeled true problems (2,656 trials) and false problems (2,649 trials) separately. Within each problem type, trials were divided into 80 design cells defined by the factorial combination of 20 participants with 2 problem size conditions (small, large) and 2 format conditions (digit, word). Afterward, two models were fitted. In the first model we employed the traditional approach where each cell is collapsed to a single mean RT. The effects of problem size and format on these mean RTs were then analyzed using a 2 × 2 analysis of variance, which relies upon null hypothesis significance testing. In addition, we computed Bayes factors using a Bayesian analysis of variance (Rouder et al., 2012); this permitted a quantitative estimation of the extent to which the observed data updated our beliefs in the underlying hypotheses that were tested with the ANOVA (including null effects).

In the second model, we fitted a shifted Wald distribution to the RTs in each design cell using the method of Anders et al. (2016). This resulted in three parameters per cell, (α, γ, θ); the effects of problems size and format on these parameters were then analyzed using traditional and Bayesian ANOVA. Technical details of the fitting algorithm can be found in the **Appendix**. All modeling was done using R (R Core Team, 2016) and the BayesFactor package (Morey and Rouder, 2015). All raw data and R scripts can be downloaded from the author's GitHub page.

### Modeling mean RTs

#### True problems

**Figure 1:**
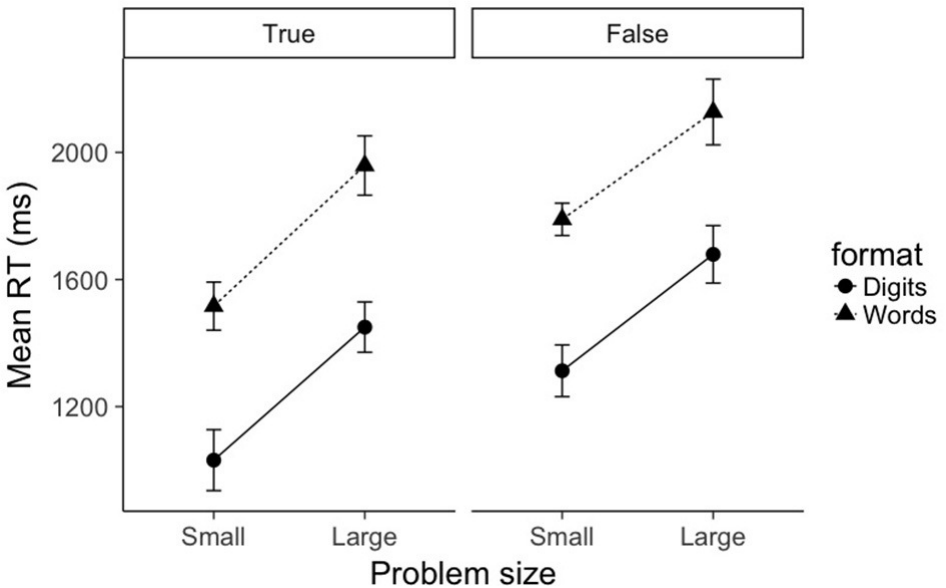
Mean RTs as a function of problem size (small, large), format (digits, words), and truth value (true, false). Error bars represent within-subject 95% confidence intervals as recommended by Morey (2008).

Mean RTs for true problems were submitted to a 2 (problem size: small, large) × 2 (format: digits, words) repeated-measures ANOVA (see left pane of Figure 1). As expected, there was a main effect of problem size, *F*_(1, 19)_ = 62.1, *p* < 0.001, $\eta_{P}^{2}=0.77$. Small problems were verified significantly faster than large problems (1,274 ms vs. 1,704 ms, respectively). There was also a main effect of format, *F*_(1, 19)_ = 219.5, *p* < 0.001, $\eta_{p}^{2}=0.92$. Digit problems were verified significantly faster than word problems (1,241 ms vs. 1,737 ms, respectively). The interaction between problem size and format was not significant (*F* = 0.243). A Bayesian ANOVA confirms these results: the best fitting model was the additive model containing factors of problem size and format ($BF_{10}=4.5\times 10^{140}$), and this model was preferred over the model containing an interaction term by a factor of 14.9. Using the convention of Jeffreys (1961), this is considered strong evidence *against* an interaction between problem size and format.

#### False problems

A similar picture emerged for false problems. Mean RTs for false problems were submitted to a 2 (problem size: small, large) × 2 (format: digits, words) repeated-measures ANOVA (see right pane of Figure 1). There was a main effect of problem size, *F*_(1, 19)_ = 46.4, *p* < 0.001, $\eta_{P}^{2}=0.71$. Small problems were verified significantly faster than large problems (1,551 ms vs. 1,903 ms, respectively). There was also a main effect of format, *F*_(1, 19)_ = 178.3, *p* < 0.001, $\eta_{p}^{2}=0.90$. Digit problems were verified significantly faster than word problems (1,496 ms vs. 1,958 ms, respectively). As with true problems, the interaction between problem size and format was not significant (*F* = 0.461). A Bayesian ANOVA confirmed that the best fitting model was again the additive model containing factors of problem size and format ($BF_{10}=2.9\times 10^{97}$), and this model was preferred over the model containing an interaction term by a factor of 13.1.

The picture that emerges from modeling only mean RTs is clear; whereas the expected effects of problem size and format are quite robust, there is strong evidence against an interaction between problem size and format.

### Modeling RT distributions

#### True problems

The distributions of RTs for true problems in each design cell [(2 (problem size: small, large) × 2 format: digits, words) × 20 (participants)] were fitted with shifted Wald distributions using the method of Anders et al. (2016) (see **Appendix**). Specifically, this method estimates values for three parameters (γ, drift rate; α, response threshold; and θ, nondecision time) for each of the 80 design cells. We will first describe the overall model fit, then separately analyze the effects of problem size and format on each of these fitted parameters.

**Figure 2:**
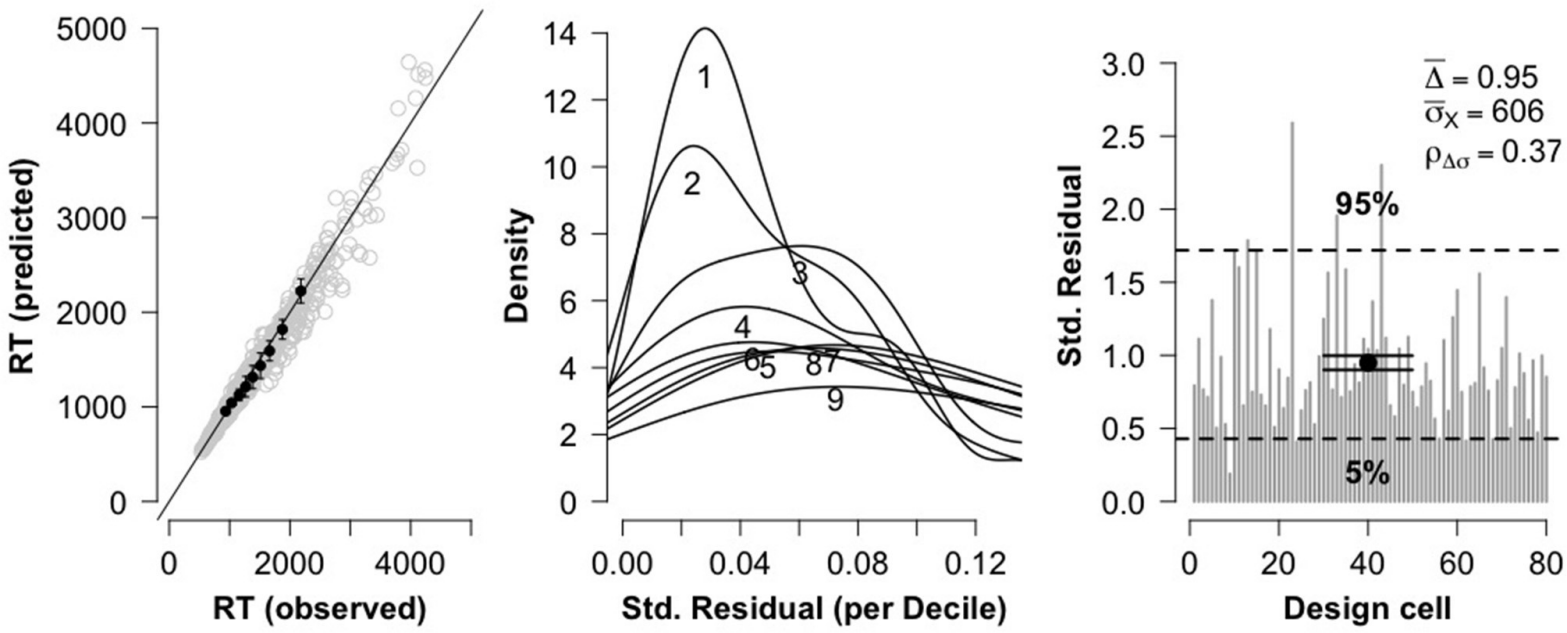
Three diagnostic plots for assessing model fit for true problems. The leftmost plot shows a QQ plot comparing observed RT deciles against model-predicted RT deciles. The center plot shows distributions of standardized residuals for each RT decile. The rightmost plot shows overall goodness of fit for each of the 80 fitted cells.

To assess model fit, three diagnostic plots were constructed (see Figure 2). The leftmost plot displays a QQ plot comparing observed RT deciles against model-predicted RT deciles. There is no obvious curvature in the plot, which is indicative of a strong model fit. The center plot displays for each RT decile the distribution of standard residuals (difference between observed data deciles and model-predicted deciles, divided by standard deviation of the distribution). The plot indicates that the residual magnitudes tend to increase with RT magnitude. Such behavior is a property of positive-skewed distributions (and in particular, simulated shifted Wald distributions; Anders et al., 2016), and is again indicative of a strong model fit. Finally, the rightmost plot displays a goodness of fit measure Δ for each of the 80 design cells, along with the average cell goodness of fit ($\bar{\Delta }$), the 5% and 95% quantile range for the Δ-values, the mean standard deviation of the observed data cells σ_*X*_, and the Pearson correlation between Δ and σ_*X*_. The plot and reported values are in line with the recommendations of Anders et al. (2016). Overall, the three diagnostic plots indicate that the data is fit quite well by the shifted Wald model.

**Figure 3:**
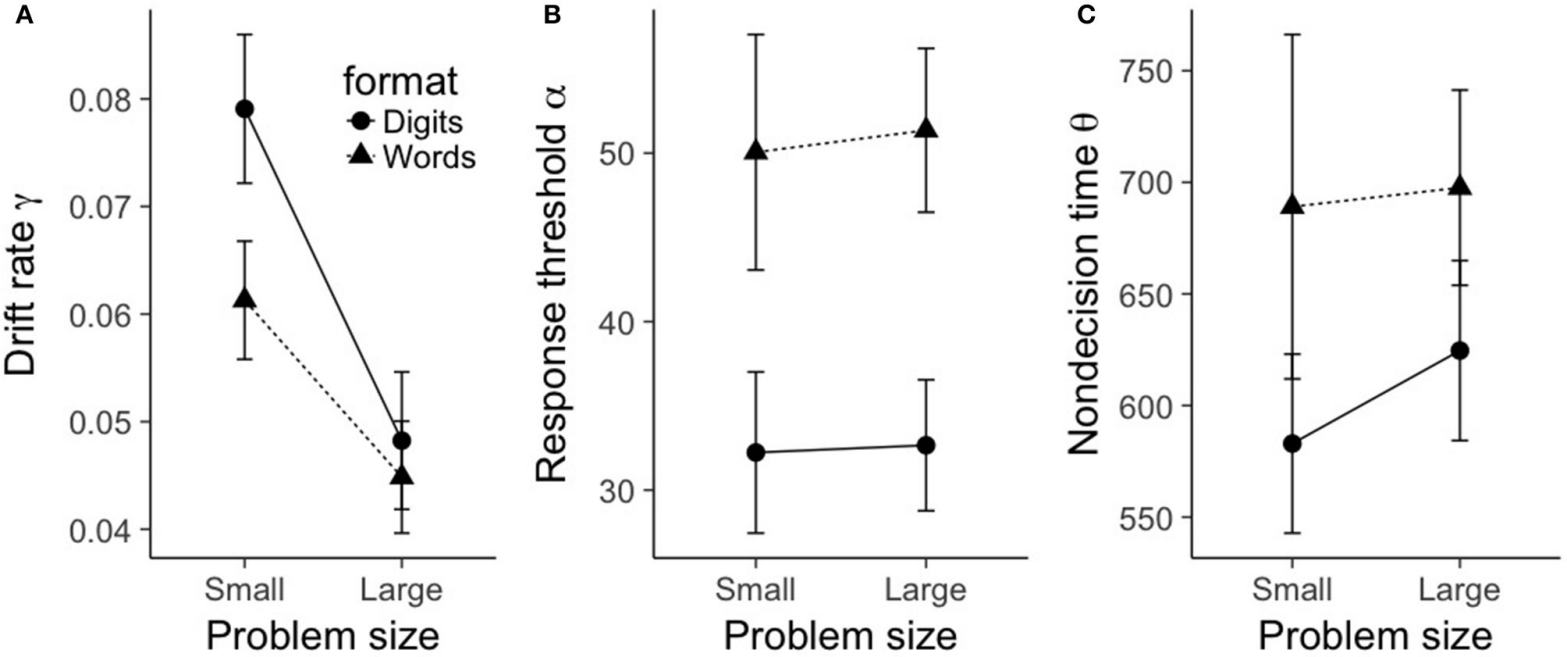
Mean shifted Wald parameters for true problems, plotted as a function of problem size (small, large), format (digits, words). **(A)** shows mean drift rate γ, **(B)** shows mean response threshold α, and **(C)** shows mean nondecision time θ. Error bars represent within-subject 95% confidence intervals as recommended by Morey (2008).

Given that the shifted Wald model is a good fit of the RT distributions, we can proceed with testing the effects of our experimental manipulations (problem size and format) on the three shifted Wald parameters. To this end, we separately submitted each parameter to a 2 (problem size: small, large) × 2 (format: digits, words) repeated-measures ANOVA. The results can be seen in Figure 3.

For drift rate γ, there was a main effect of problem size, *F*_(1, 19)_ = 71.6, *p* < 0.001, $\eta_{p}^{2}=0.79$. As can be seen in Figure 3A, small problems had a significantly larger drift rate (0.07) compared to large problems (0.05). There was also a main effect of format, *F*_(1, 19)_ = 11.1, *p* = 0.003, $\eta_{p}^{2}=0.37$. Digit problems exhibited a significantly larger drift rate (0.064) than word problems (0.053). Finally, there was a significant interaction between problem size and format, *F*_(1, 19)_ = 7.3, *p* = 0.014, $\eta_{p}^{2}=0.28$. As is evident from Figure 3A, the effect of format on drift rate was restricted to small problems. A Bayesian ANOVA gives moderate support for this pattern of results, as the best fitting model included the interaction between problem size and format ($BF_{10}=6.27\times 10^{8}$), and this model was preferred over the additive-only model by a factor of 3.15.

For response threshold α, there was only a main effect of format, *F*_(1, 19)_ = 62.7, *p* < 0.001, $\eta_{p}^{2}=0.77$. As can be seen in Figure 3B, word problems had a significantly larger mean response threshold (50.7) than digit problems (32.4). No other terms in the ANOVA model were significant (all *F*-values less than 0.86). A Bayesian ANOVA yielded a best fitting model that included only a term for format ($BF_{10}=2.01\times 10^{7}$), and this model was preferred over a model that contained an additional term for problem size by a factor of 4.25.

A similar picture emerges with nondecision time θ; again, there was only a main effect of format, *F*_(1, 19)_ = 11.3, *p* = 0.003, $\eta_{p}^{2}=0.37$. As can be seen in Figure 3C, word problems had a significantly longer mean nondecision time (693 ms) than digit problems (604 ms). No other terms in the ANOVA model were significant (all *F*-values less than 0.47). A Bayesian ANOVA yielded a best fitting model that included only a term for format (*BF*_10_ = 40.4), and this model was preferred over a model that contained an additional term for problem size by a factor of 2.65.

#### False problems

**Figure 4:**
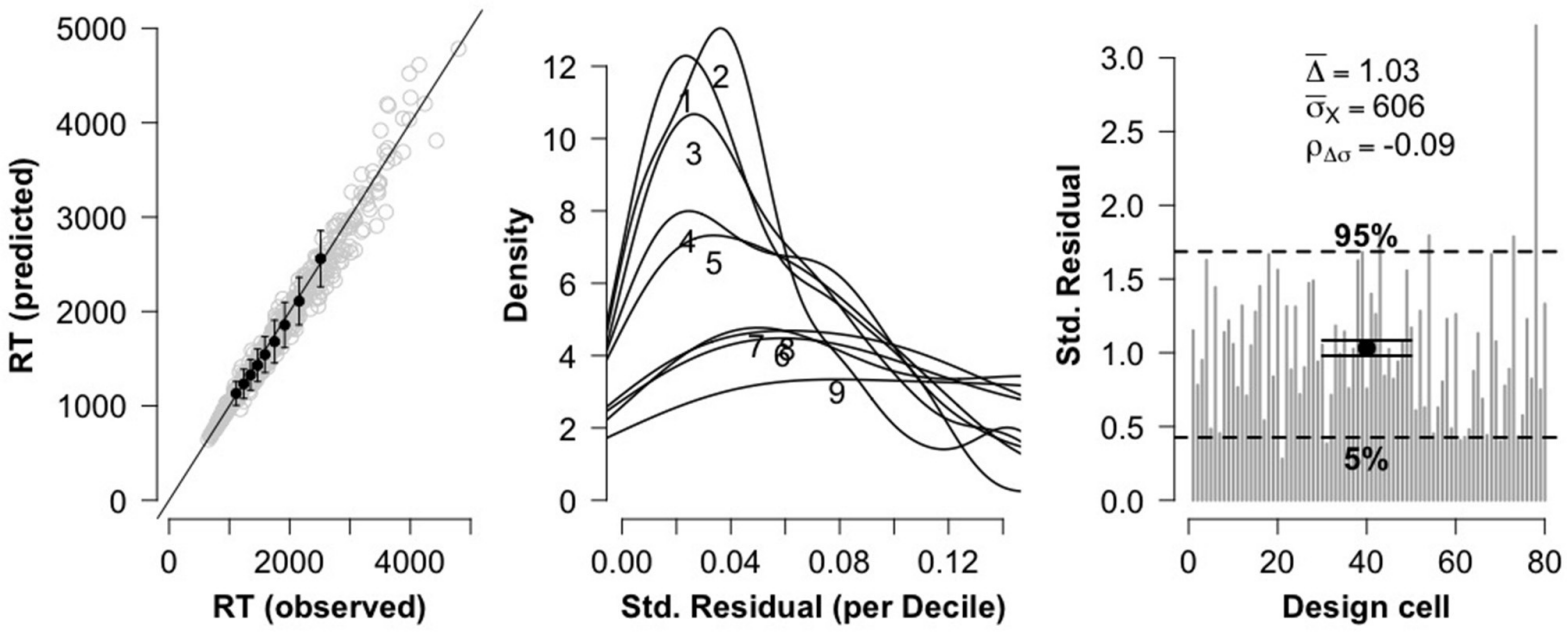
Three diagnostic plots for assessing model fit for false problems. The leftmost plot shows a QQ plot comparing observed RT deciles against model-predicted RT deciles. The center plot shows distributions of standardized residuals for each RT decile. The rightmost plot shows overall goodness of fit for each of the 80 fitted cells.

As with true problems, the distributions of RTs for false problems in each design cell [2 (problem size: small, large) × 2 (format: digits, words) × 20 (participants)] were fitted with shifted Wald distributions. As can be seen in Figure 4, the three diagnostic plots are again in line with the recommendations of Anders et al. (2016), thus indicating that these data are fit quite well by the shifted Wald model.

**Figure 5:**
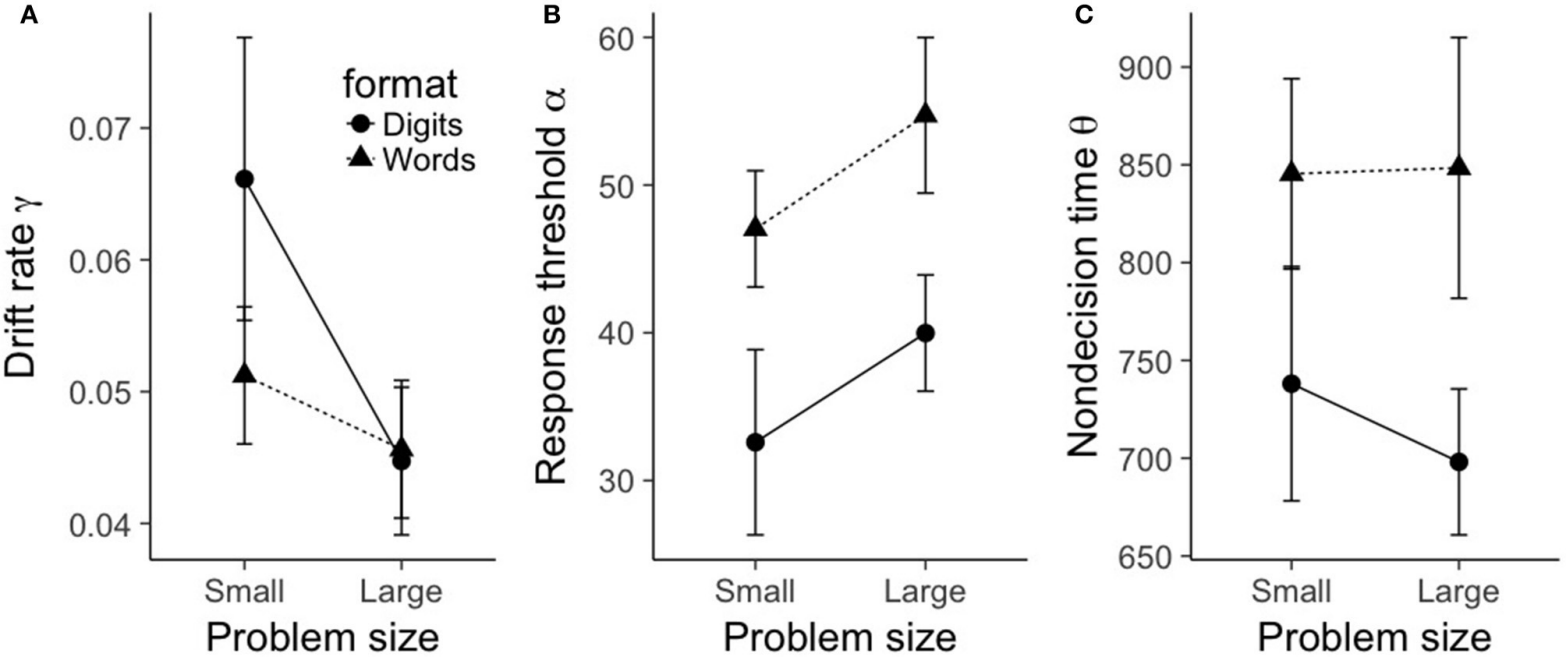
Mean shifted Wald parameters for false problems, plotted as a function of problem size (small, large), format (digits, words). **(A)** shows mean drift rate γ, **(B)** shows mean response threshold α, and **(C)** shows mean nondecision time θ. Error bars represent within-subject 95% confidence intervals as recommended by Morey (2008).

Given the acceptable fit of the shifted Wald model, we submitted each parameter to a 2 (problem size: small, large) × 2 (format: digits, words) repeated-measures ANOVA. The results can be seen in Figure 5. For drift rate γ, there was a main effect of problem size, *F*_(1, 19)_ = 17.0, *p* < 0.001, $\eta_{p}^{2}=0.47$. As can be seen in Figure 5A, small problems had a significantly larger drift rate (0.06) compared to large problems (0.045). The main effect of format was not statistically significant, *F*_(1, 19)_ = 3.78, *p* = 0.07, but there was a significant interaction between problem size and format, *F*_(1, 19)_ = 5.86, *p* = 0.026, $\eta_{p}^{2}=0.23$. Figure 5A reveals a similar picture to the situation we saw with true problems; the (albeit marginal) effect of format on drift rate was again restricted to small problems. A Bayesian ANOVA indicated that the best fitting model included the interaction between problem size and format (*BF*_10_ = 338), and this model was preferred over the model with two main effects (problem size and format) by a factor of 2.90.

For response threshold α, we found a significant main effect of problem size, *F*_(1, 19)_ = 10.3, *p* = 0.005, $\eta_{p}^{2}=0.35$. As can be seen in Figure 5B, large problems exhibited a larger response threshold (47.4) than small problems (39.8). We also saw a main effect of format, *F*_(1, 19)_ = 24.8, *p* < 0.001, $\eta_{p}^{2}=0.57$; word problems exhibited a larger response threshold (50.9) than digit problems (36.3). The interaction between format and problem size was not significant, *F*_(1, 19)_ = 0.008, *p* = 0.93. A Bayesian ANOVA indicated that the best fitting model was the model with two main effects (problem size and format) (*BF*_10_ = 701, 677), and this model was preferred over a model that contained an additional interaction term by a factor of 3.22.

The results for nondecision time θ mirrored those for true problems. As before, there was only a main effect of format, *F*_(1, 19)_ = 20.8, *p* < 0.001, $\eta_{p}^{2}=0.52$. As can be seen in Figure 5C, word problems exhibited a significantly longer nondecision time (847 ms) than digit problems (718 ms). No other terms in the ANOVA model were significant (all *F*-values less than 1.6). A Bayesian ANOVA yielded a best fitting model that included only a term for format (*BF*_10_ = 3, 039), and this model was preferred over a model that contained an additional term for problem size by a factor of 3.60.

## Discussion

The purpose of the present study was to use a single boundary accumulator model (the shifted Wald distribution) to investigate the independence of encoding and calculation processes in mental arithmetic. Previous research has presented evidence in favor of two competing models: an additive model (Dehaene, 1992; McCloskey, 1992), where encoding processes are isolated from calculation processes, and an interactive model (Campbell and Clark, 1988; Campbell and Epp, 2004), where encoding processes interact with calculation processes. Past studies have attempted to decide between these models by looking for an interaction between the effects of format and problem size on mean RTs. In this paper, we extended this approach and fit a shifted Wald model (Anders et al., 2016) to the distribution of RTs in each experimental condition. This resulted in a collection of three parameters (drift rate, response threshold, and nondecision time) on which we could then test the effects of format and problem size. We found that drift rate was affected by both problem size and format, but response threshold and nondecision time were generally affected only by format. As we will explain below, such results (in particular, the effect of format on drift rate), favor an interactive model of mental arithmetic (e.g., Campbell, 1994).

One of the primary advantages of modeling *distributions* of RTs (compared to analyzing mean RTs alone) is that this modeling approach provides a substantial increase in measurement resolution. To see this, consider that in our experiment, we found no interaction between format and problem size when restricting our attention to mean RTs. If we limited our analysis to this null effect on mean RTs, it would seem that we have found support for an additive model, where encoding and calculation are independent from each other. Moreover, this result would not likely be due to a Type II error (i.e., a false negative), where our null effect would be simply the result of failing to find a significant interaction due to inherently low power. On the contrary, we computed a Bayesian analysis of variance which indicated a Bayes factor of 13.1 in favor of the additive model. This means that after observing the data, we should update the ratio of our belief in the additive model (over the interactive model) by a factor of 13.1, which is considered fairly strong evidence (Jeffreys, 1961). This is a surprising result, as our results certainly do not match the previous findings of Campbell and colleagues (e.g., Campbell and Fugelsang, 2001), who consistently find strong format by problem size interactions.

However, we saw a different picture emerge when we analyzed the *distributions* of RTs in each experimental condition. First, we found that problem size and format interacted to impact drift rate. Specifically, drift rates decreased for as problem size increased. Also, when restricting to small problems, drift rates were larger for digit problems compared to word problems, but this advantage disappeared for large problems. If we assume that our estimated drift rates reflect the cognitive processes related to calculation, the fact that format affects drift rate implies that our format manipulation has a direct impact on calculation. This supports an interactive architecture of mental arithmetic (Campbell, 1994).

In addition to the various effects on drift rate, we saw format effects on response threshold α and nondecision time θ. Compared to digit problems, problems presented in word format required more accumulated information before response initiation (i.e., larger response threshold α). Similarly, word problems also exhibited larger nondecision times θ than digit problems. One possible explanation is that encoding costs might be incurred when using less familiar word-based stimuli compared to the more familiar digit stimuli. At present, this is speculative, as the format effects on θ reflect a general cost of format on processes external to signal accumulation (not just encoding). This leaves open the possibility that format may affect only encoding processes, only response processes, or both. The second option is unlikely, as all existing models of mental arithmetic predict that format affects encoding processes. However, it is not currently clear whether format additionally affects the later processes involved in responding. Several recent studies have indicated that manipulations of number encoding can feed forward into the response phase, at least in simple number decision tasks (e.g., Santens and Verguts, 2011; Faulkenberry et al., 2016; Sobel et al., 2016, 2017). As such, the third option remains viable; hopefully future studies can further address the effects of format on response processes in mental arithmetic.

We also found an interesting, yet unexpected interaction between the effects of format and problem size on drift rate. Specifically, small problems exhibited a large format effect, where drift rates were significantly larger for digits than for words. However, this effect disappeared for large problems. While we can only speculate at this time, this interaction could be due to a difference in solution strategies employed for small and large problems. Indeed, small problems tend to use long term memory retrieval (Ashcraft, 1992; Campbell and Xue, 2001), whereas larger problems tend to use nonretrieval strategies (LeFevre et al., 1996). Our results could reflect the idea that for retrieval-based calculation processes, Arabic digits result in better quality of stimulus information than word format problems (perhaps because the Arabic digit format better matches the way in which such small arithmetic facts were originally learned). However, such format effects could be erased when nonretrieval strategies are used, most likely because the underlying cognitive processes are more complex in this case and are not completely reflected by drift rate. At this point, this is an excellent open question for future research.

We think that these results are an important first step for a new approach to studying problems in mathematical cognition. By fitting the distribution of RTs instead of collapsing all data to a single measure, we were able to capture behavioral phenomena that we would have simply missed by focusing on the traditional mean RT measures. In other words, using accumulator models of RTs results in better measurement fidelity than that obtained by mean RTs, a perspective long advocated in the field of mathematical psychology (e.g., Luce, 1986). A second advantage of our modeling approach is that we were able to get more direct measures of the cognitive processes involved in mathematical decision making. We hope that other researchers will extend this approach to a more general framework for building and testing theories of the cognitive processes involved in mathematical thinking.

We opted to use the shifted Wald distribution (a single boundary accumulator model) in this study, but there is no reason that future studies could not use other accumulator models, such as the diffusion model of Ratcliff and colleagues (Ratcliff and McKoon, 2008; Ratcliff et al., 2016). One reason we opted for the shifted Wald distribution is because the diffusion model is difficult to fit when participants make few errors (Anders et al., 2016). On the other hand, the shifted Wald distribution is perfectly suited to such tasks. The only concession that we had to make is that we had to remove errors from analysis, which prevents us from being able to assess speed-accuracy tradeoffs. Clearly, experiments designed to test predictions about speed-accuracy tradeoffs should consider the more general diffusion framework, which allows modeling both correct and incorrect responses. Another advantage to the shifted Wald distribution is that it requires a relatively small number of trials in each experimental condition. We were able to fit the shifted Wald distribution to our participants' data with 72 trials per condition. Anders et al. (2016) showed that shifted Wald parameters can be recovered quite well for as few as 50 observations per condition.

We should note that using the shifted Wald distribution to theorize about cognitive processes should be done with care. While it is tempting to make direct associations between the drift rate, response threshold, and nondecision time of the shifted Wald model and the similarly-defined drift rate, response threshold, and nondecision time of the diffusion model, such a mapping is not directly obvious. For example, Matzke and Wagenmakers (2009) simulated data using a two-boundary diffusion process, then subsequently fit the data with a single-boundary shifted Wald model. They found that the recovered shifted Wald parameters did not correspond uniquely with the diffusion parameters used to simulate the data. Thus, it is not entirely clear that shifted Wald parameters should be interpreted the same way that diffusion parameters are interpreted. However, Anders et al. (2016) notes that in situations like the ones modeled by Matzke and Wagenmakers (2009), the shifted Wald would exhibit a poor model fit anyway. Thus, since our data exhibited a reasonably good model fit, we are cautiously confident in our cognitive interpretations of our obtained shifted Wald parameters. Of course, more research is needed in order to better understand when (and how) the shifted Wald distribution can be used as a cognitive process model.

Finally, we note that the choice of task is important to studies in mathematical cognition. A verification task was ideal for the present study, as it framed our mental arithmetic task as a two-choice decision task, which is standard in studies involving accumulator models of RTs. However, it is important to note a verification task might not necessarily be the best reflection of the processes involved in arithmetic. One reason is that decisions might not always be derived from the same calculation processes involved in production. For example, on some problems, participants could rely on a shortcut strategy for detecting false problems, such as knowing that the outcome can only be odd if only one of the addends is odd. As such, the processes involved in this decision would be quite different from the processes involved if the problem was solved by first calculating the answer, then comparing the calculated answer to the one presented. For future studies, it will be important to consider this type of modeling for production tasks as well. We think a single-boundary accumulator model is ideal for this.

In summary, we used a single boundary accumulator model (the shifted Wald distribution) to fit RT distributions in an arithmetic verification task. While we found no interaction between problem format and problem size on mean RTs, we did find that format directly affected drift rate. Thus, we conclude that format affects are not isolated to the encoding stage (as predicted by additive models, e.g., Dehaene, 1992; McCloskey, 1992). Instead, our data supports an interactive model of arithmetic processing (Campbell, 1994), where the effects of problem format extend beyond the encoding stage to have direct impacts on the processes involved in calculation.

## Ethics statement

This study was carried out in accordance with the recommendations of the APA ethics code, with written informed consent from all subjects. All subjects gave written informed consent in accordance with the Declaration of Helsinki. The protocol was approved by the Institutional Review Board at Tarleton State University.

## Author contributions

TF designed and conducted the experiment, analyzed and modeled the data, and wrote the manuscript.

### Conflict of interest statement

The author declares that the research was conducted in the absence of any commercial or financial relationships that could be construed as a potential conflict of interest. The reviewer KL and handling Editor declared their shared affiliation, and the handling Editor states that the process nevertheless met the standards of a fair and objective review.

## Footnotes

^1^Though they did not report any inferential statistics for this specific interaction, they did report that problem size did not interact with any other variable (including format), with all *p*-values greater than 0.12 (p. 356).

## Appendix

### Fitting the shifted wald model

The approach used for fitting the shifted Wald model in this paper is similar to that first reported in Anders et al. (2016). The idea is to map the raw data onto three parameter estimates $\hat{\alpha }$, $\hat{\gamma }$, $\hat{\theta }$ that produce predicted RTs that are as close as possible to the actual RTs. This is done in the following manner.

Let ${\left\{X_{i}\right\}}_{i=1}^{M}$ be a set of *M* independent observations; in our case, this would be a set of RTs for *M* trials. Let $\beta =1/\left(\hat{\alpha }\hat{\gamma }\right)$. Given a value for β, one can iteratively calculate maximum likelihood estimators (MLEs) for $\hat{\theta }$ and $\hat{\alpha }$ (Nagatsuka and Balakrishnan, 2013). To begin, we first compute a “seed”:

(A1) $${\hat{a}}_{0}^{*}=\sqrt{\frac{{\left(\bar{X}-X_{\text{min}}\right)}^{3}}{\frac{1}{M}\sum_{k=1}^{M}{\left(X_{k}-\bar{X}\right)}^{2}}}$$

Then, using this seed, we can obtain the MLEs for $\hat{\theta }$ and $\hat{\alpha }$ using the equations

(A2) $$\hat{\theta }=X_{\text{min}}-{\hat{a}}_{0}^{2}{\int_{0}^{\infty }\left(1-F[z;\hat{\beta },1,0]\right)}^{M}dz$$

and

(A3) $$\hat{\alpha }={\left(\frac{1}{M}\sum_{k=1}^{M}{\left(X_{k}-\hat{\theta }\right)}^{-1}-{\left(\bar{X}-\hat{\theta }\right)}^{-1}\right)}^{-1/2},$$

where *F*(·) is the cumulative distribution function of the shifted Wald distribution (Equation 1). Specifically, the initial seed ${\hat{\alpha }}_{0}^{*}$ obtained in Equation (A1) is input into Equation (A2) in place of ${\hat{\alpha }}_{0}$, which then produces an estimate ${\hat{\theta }}^{*}$. We then compute ${\hat{\alpha }}_{0}$ via Equation (A3) by setting $\hat{\theta }={\hat{\theta }}^{*}$. Afterward, this value of ${\hat{\alpha }}_{0}$ is used to compute $\hat{\theta }$ and then $\hat{\alpha }$ via Equations (A2) and (A3), respectively. Finally, $\hat{\gamma }$ can be computed easily as $\hat{\gamma }=1/\beta \hat{\alpha }$.

From these MLEs ($\hat{\alpha }$, $\hat{\gamma }$, $\hat{\theta }$), one uses the Wald pdf (Equation 1) to calculate a set of *M* predicted quantiles ${\left\{Q_{k}^{P}\right\}}_{k=1}^{M}$ which can then be compared to the observed quantiles (from our data) ${\left\{Q_{k}^{O}\right\}}_{k=1}^{M}$ via an *l*_1_-norm:

(A4) $$\begin{array}{l} \|Q^{P}-Q^{O}{\|}_{1}=\sum_{k=1}^{M}|Q_{k}^{P}-Q_{k}^{O}|. \end{array}$$

Thus, for every chosen value of β, we obtain a deviance measure $||Q^{P}-Q^{O}|{|}_{1}.$ This procedure is repeated over a plausible range of values for β (e.g., 0.01 < β < 0.99), and the parameter set that minimizes $||Q^{P}-Q^{O}|{|}_{1}$ is chosen as the fit.
